# Let’s Talk About BiTEs and Other Drugs in the Real-Life Setting for B-Cell Acute Lymphoblastic Leukemia

**DOI:** 10.3389/fimmu.2019.02856

**Published:** 2019-12-20

**Authors:** Dalma Deak, Cristina Pop, Alina-Andreea Zimta, Ancuta Jurj, Alexandra Ghiaur, Sergiu Pasca, Patric Teodorescu, Angela Dascalescu, Ion Antohe, Bogdan Ionescu, Catalin Constantinescu, Anca Onaciu, Raluca Munteanu, Ioana Berindan-Neagoe, Bobe Petrushev, Cristina Turcas, Sabina Iluta, Cristina Selicean, Mihnea Zdrenghea, Alina Tanase, Catalin Danaila, Anca Colita, Andrei Colita, Delia Dima, Daniel Coriu, Hermann Einsele, Ciprian Tomuleasa

**Affiliations:** ^1^Department of Hematology, Iuliu Hatieganu University of Medicine and Pharmacy, Cluj-Napoca, Romania; ^2^Department of Hematology, Ion Chiricuta Clinical Cancer Center, Cluj-Napoca, Romania; ^3^Department of Pharmacology, Faculty of Pharmacy, Iuliu Hatieganu University of Medicine and Pharmacy, Cluj-Napoca, Romania; ^4^Research Center for Advanced Medicine, Iuliu Hatieganu University of Medicine and Pharmacy, Cluj-Napoca, Romania; ^5^Research Center for Functional Genomics and Translational Medicine, Iuliu Hatieganu University of Medicine and Pharmacy, Cluj-Napoca, Romania; ^6^Department of Hematology, Fundeni Clinical Institute, Bucharest, Romania; ^7^Department of Hematology, Grigore T. Popa University of Medicine and Pharmacy, Iasi, Romania; ^8^Department of Hematology, Regional Institute of Oncology, Iasi, Romania; ^9^Department of Stem Cell Transplantation, Fundeni Clinical Institute, Bucharest, Romania; ^10^Department of Pediatrics, Carol Davila University of Medicine and Pharmacy, Bucharest, Romania; ^11^Department of Hematology, Coltea Hospital, Bucharest, Romania; ^12^Department of Hematology, Carol Davila University of Medicine and Pharmacy, Bucharest, Romania; ^13^Department of Internal Medicine II, University Hospital Wurzburg, Würzburg, Germany; ^14^Department of Hematology/Research Center for Functional Genomics and Translational Medicine, Iuliu Hatieganu University of Medicine and Pharmacy, Cluj-Napoca, Romania

**Keywords:** blinatumoman, acute lymphoblastic leukemia, bridge-to-transplant, real life setting, bispecific antobodies

## Abstract

**Background:** Therapy for acute lymphoblastic leukemia (ALL) are currently initially efficient, but even if a high percentage of patients have an initial complete remission (CR), most of them relapse. Recent data shows that immunotherapy with either bispecific T-cell engagers (BiTEs) of chimeric antigen receptor (CAR) T cells can eliminate residual chemotherapy-resistant B-ALL cells.

**Objective:** The objective of the manuscript is to present improvements in the clinical outcome for chemotherapy-resistant ALL in the real-life setting, by describing Romania's experience with bispecific antibodies for B-cell ALL.

**Methods:** We present the role of novel therapies for relapsed B-cell ALL, including the drugs under investigation in phase I-III clinical trials, as a potential bridge to transplant. Blinatumomab is presented in a critical review, presenting both the advantages of this drug, as well as its limitations.

**Results:** Bispecific antibodies are discussed, describing the clinical trials that resulted in its approval by the FDA and EMA. The real-life setting for relapsed B-cell ALL is described and we present the patients treated with blinatumomab in Romania.

**Conclusion:** In the current manuscript, we present blinatumomab as a therapeutic alternative in the bridge-to-transplant setting for refractory or relapsed ALL, to gain a better understanding of the available therapies and evidence-based data for these patients in 2019.

## B-Cell All From Diagnosis to CR1, Relapse, and CR2

The use of combination chemotherapy for B-cell ALL has improved the therapeutic ratio for these patients, reported to achieve complete remission (CR) rates of 80%. Half of the patients have long-term disease control with consolidation and maintenance chemotherapy, but 10–15% of them develop primary refractory disease (1, 2). Many more patients ultimately relapse and only 20–30% of them achieve a second CR (CR2) with standard salvage chemotherapy (3). In 2017, the Food and Drug Administration (FDA) in the US has approved inotuzumab ozogamycin (InO) and blinatumomab for relapsed/refractory (R/R) B-cell ALL (4–6) and in 2018 tisagenlecleucel (TISA, formally CTL09) (7–9). B-cell ALL blasts express CD22, rendering them excellent targets for InO (10). Similarly, blinatumomab is a bispecific T-cell engager antibody construct that allows cytotoxic T-cells to recognize and eliminate CD19-expressing B cells (11). In the present manuscript, we present new immunotherapy-based therapy selection for R/R ALL. InO, blinatumomab and tisagenlecleucel are preferred over traditional chemotherapy regimens for R/R ALL. However, trials comparing different immunotherapeutic options have not been conducted.

Options now include a diverse selection of small-molecule–targeted inhibitors, monoclonal antibodies against tumor antigens with and without attached toxic cargoes, and several novel immunotherapies (12, 13). The latter category has gained particular traction in recent years. Successful development efforts include immune checkpoint inhibitors to counteract tumors' immune-inhibitory signals (14, 15), reprogramming of T cells to attack tumors with chimeric Ag receptors and, finally, bispecific Abs (BiTEs) that promote immune synapse formation between immune effectors and malignant cells (16). A very successful approach has been the development of BiTEs, fusion proteins with specificity for two antigens functioning as activating magnets between effector and tumor cells (11, 17, 18). Only one BiTE, blinatumomab, currently has regulatory approval for clinical use, but the established proof of principle is fueling extensive efforts to expand the approach to additional tumor and effector cell types. Following FDA approval in the US, the drug received accelerated approval because of significant rates of objective responses in a disease with widely unmet medical needs. Indeed, this was quickly expanded to a full approval for adults and children to treat R/R B-cell precursor ALL Philadelphia chromosome-negative or positive in July 2017 (19, 20). Still, this drug has some unmet needs, mostly related to the real-life setting. In the current manuscript, we describe both the impressing advantages of the molecule, as well as it's unmet need, presenting Romania's experience as a real-life scenario.

The treatment of ALL reached unprecedented achievements over the past few years, particularly in the pediatric setting, where the long-term overall survival rate reached 80% (21, 22). Furthermore, recent data predicts that the cure rate will increase to 90% shortly (23–26). Still, the cure rate of ALL in adults remains unsatisfactory and the pediatric experience is not reported in adults, where the optimistic survival barely reaches 35–40% for patients younger than 60 years, and <10% for those older than 60 years (27–29).

Risk stratification allows physicians to adequately determine initial treatment regimen as well as when to consider allogeneic stem cell transplant (SCT) (30–32). Age is the central factor to consider during risk stratifying of patients, with an increased age being correlated with worsening prognosis (33). Patients over the age of 60 have particularly poor outcomes, with only 10–15% long-term survival (34, 35). Since prognostic factors have been clearly defined in recent years, the clinical management based on cytogenetic and molecular biology allows physicians to easily stratify patients. Therefore, high-risk patients are those with pro-B phenotype, Philadelphia (Ph)-positive ALL, *t*(4, 11) karyotype, hypodiploidy, high WBC at the diagnosis defined as more than 30 × 10^9^ WBC, as well as achievement of complete remission in more than 45 days following initiation of treatment (36–44).

For ALL, CR is usually defined as <5% blasts in the bone marrow, normal maturation of all cellular components in the bone marrow, no extramedullary disease (e.g., central nervous system, soft tissue disease), ANC (absolute neutrophil count) of at least 1,000/μL, platelets more than 100,000/μL and transfusion independent patients.

Following CR1, treatment options include either consolidation and maintenance chemotherapy or allogeneic SCT for eligible patients (8, 29, 45–49). A patient with Ph-positive ALL is an absolute indication of allogeneic SCT following CR1, considering that matched-sibling allogeneic SCT can increase long-term survival to 35–55% (29, 38, 50). These results were also confirmed by an interim analysis of the Acute Leukemia Working Party of the European Bone Marrow Transplantation (EBMT) Society. Still, the possibility of finding a matched donor remains very limited, but the option of a haploidentical donor remains a viable option; as Ciurea et al. pointed out (51–53).

Timely follow-up with patients in CR1, after chemotherapy or after an allogeneic STC is based on measurable disease (MRD) analysis. This is of great importance in both acute leukemia as well as in other hematological malignancies (54–56). MRD is an independent risk factor for decreased relapse-free survival (RFS) and shorter overall survival (OS) (31, 57, 58). Allogeneic SCT in the case of standard-risk adults has an ambiguous role with the advent of MRD as a prognostic marker capable of easily restratifying patients to high-risk, thus rendering them candidates for SCT. In patients with molecularly undetectable leukemia, there is no survival advantage conferred by SCT compared to standard chemotherapy. However, for positive MRD, SCT has been linked with improved relapse-free survival. Nowadays, two 8-color tube tests reached a specificity and a sensitivity comparable to PCR-based methods. Markers were selected using novel software tools and principal component analysis. Protocols were designed to acquire at least 4 million cells. The following markers are considered indispensable: CD81, CD38, CD66c+, CD123, CD73+, and CD30. Further improvements designed to surpass immunophenotype changes induced by blinatumomab or T CAR cells proposed the addition of CD24 and CD22 in 10- color tubes. There were various attempts to design detection panels for B-ALL MRD that are more specific and sensitive than current diagnostic tools. However, all of them faced the challenge of finding surface molecules that would accurately distinguish normal, regenerative bone marrow from leukemic persistent or relapsed parts. To detect leukemia-associated aberrant phenotypes, the first step is to fully characterize normal B-cell precursors. B- ALL blasts resemble normal blasts regarding most of the commonly analyzed markers. Nevertheless, an aberrant immunophenotype can be detected in 95% of pediatric ALL thus allowing efficient MRD detection. During normal B cell maturation, CD34 is first down-regulated, together with TdT, followed by CD10 and CD38, while the expression of CD45, CD21, and CD22 is up-regulated. Most aberrancies are related to the co-, over- or underexpression of CD10, TdT, CD38, CD34, CD20, and cross lineage myeloid expression, while aberrant T cell antigen expression is less frequent. For T-cell ALL, in the bone marrow there is a low level of surface molecules specific for myeloid cell or for B cells. Flow cytometry MRD detection is based on the asynchronous expression of antigens in comparison with a normal maturation pattern.

Another matter of concern is that normal bone marrow can contain minor populations of cells with atypical maturation patterns. With the help of sensitive techniques, these populations could be identified in the regenerative bone marrows of cancer patient's post-chemotherapy for solid tumors. Maturation patterns in regenerating bone marrow after ALL treatment are influenced by the intensity of the therapeutic regimen, which stimulates the development of a specific subpopulation of cells and causes modifications in the expression of surface molecules. This can cause difficulties in the interpretation of data and generate false-positive results. False-negative results are also reported, mainly due to uneven distribution of leukemic cells and to clonal evolution, which means that the immunophenotype at diagnosis is not conserved during follow-up. Through repeated testing from different sampling sites and the use of a comprehensive follow-up panel, these problems are overcome. Nonetheless, it is still recommended to use both flow cytometry and PCR for MRD detection in spite of reliable molecular markers such as *BCR-ABL*. The discordances, regarding overestimating MRD by PCR, may be caused by the mutation with multilineage involvement (B, T, myeloid, and/or erythroid).

**Figure 1:**
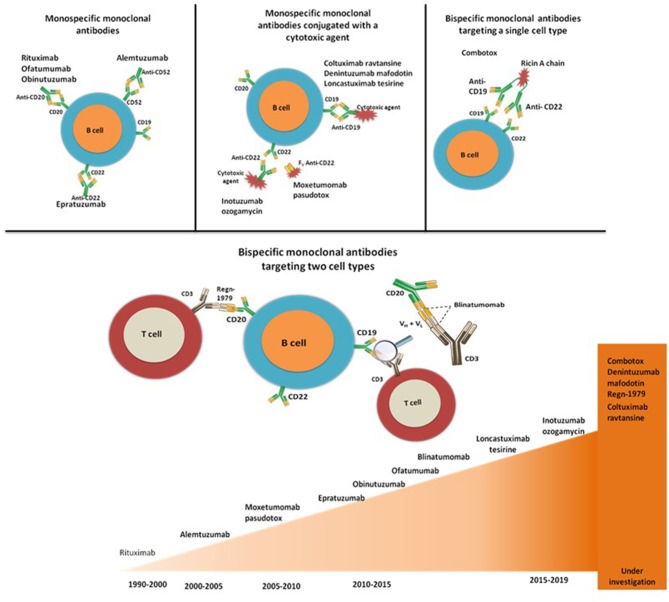
Evolution of monoclonal antibody-based for B-cell ALL.

Current methods of MRD detection through flow cytometry are still under improvement. It is still under debate which independent marker or combination of markers is more reliable. Thus, MRD is a prognostic marker that reclassifies patients to the high-risk category for SCT transplants. When analyzing the risk factors in CR1 patients that have undergone an allogeneic SCT vs. standard chemotherapy, for patients with positive MRD, allogeneic SCT was associated with increased relapse-free survival (RFS) (59–61), but for the ones with negative MRD, no survival benefit was reported between SCT and standard chemotherapy. As follows, even if the role of allogeneic SCT in the consolidation phase after CR1 is not superior to standard chemotherapy, it should be the first treatment-of-choice for relapsed cases. Tavernier et al. report that a transplant is superior to standard chemotherapy after CR2 (62), with patients that had an SCT from a sibling donor having a higher 5-year survival in comparison with the ones with an SCT from a matched unrelated donor (MUD) (63, 64). Standard chemotherapy has limited results in the second complete remission (CR2) ALL patients, where an allogeneic SCT is the main viable therapeutic option for long term survival. Current efforts in advancing the treatment of ALL focus on a more targeted approach. Immunotherapy is a broad and promising field able to provide alternative therapeutics for cancer patients, especially in the relapsed/refractory (R/R) setting where standard chemotherapy has limited results (64). As follows, small molecules and monoclonal antibodies brought forward a new perspective for salvage therapy (Figure 1).

These alternative options of immunotherapy have been validated in hematological malignancies, especially in B-ALL. In ALL, leukemic blasts express the following surface antigens: CD19, CD20, CD22, CD33, and CD52. Therefore, various monoclonal antibody-based drugs are able not only to selectively target these antigens and the malignant cells, but also to minimize off-target toxicity. Treatment with monoclonal antibodies in adult ALL is already the standard-of-care in some cases. For instance, in B-ALL the combination of rituximab with chemotherapy increases significantly the OS (65, 66). In R/R ALL, blinatumomab and inotuzumab ozogamycin (InO) are either under investigation in phase III clinical trials or they have recently been approved by the Food and Drug Administration (FDA) in the US. However, these agents bring forward new alternatives for the management of ALL, redefining the standards-of-treatment and the options for different risk subsets, as further presented.

## Anti-CD20 Immunotherapy

CD20 is a B cell differentiation antigen widely expressed during B cell development from early pre-B cells until mature B lymphocytes (67, 68). In the blood physiology, CD20 regulates cell cycle initiation and possibly other functions, such as calcium channel modulator (69–71). CD20 is expressed in 40–50% of all precursor lymphoblasts. Expression correlated to *de novo* adult precursor B-ALL appears to also be associated with a poor prognosis, particularly in younger patients (72–74). As follows, various monospecific monoclonal antibodies have been investigated and approved by the FDA for the treatment of B-ALL, as seen in Table 1 (75–80).

**Table 1:** Romanian experience with the use of blinatumomab for B-cell ALL until May 2019.

**Case number**	**Sex**	**Age**	**Phenotype**	**CRS**	**SCT**	**Treatment protocol**	**MRD after first line**	**OS months**	**OS censor**	**RFS months**	**RFS censor**	**Fusion protein**	**Cytogenetics**
1	M	24	Common ALL	NA	MUD	Hyper CVAD	Positive	68	0	44	1	NA	NA
2	M	49	Pro B-ALL	CRS	Haplo	EORTC	Positive	NA	NA	NA	NA	NA	NA
3	F	39	B-ALL	NA	NA	GRAALL	Negative	1	1	1	0	BCR-ABL1	NA
4	M	18	B-ALL	CRS	NA	BFMALL200	Negative	39	0	32	1	E2A-PBX1	Normal karyotype
5	M	25	Common ALL	NA	NA	PETHEMAALL93	Negative	20	0	5	1	NA	del 6q21
6	F	42	Pro B-ALL	NA	MUD	PETHEMAALL93	Positive	29	0	2	1	MLL-AF4	Hyperdiploid karyotype
7	F	48	Common ALL	NA	NA	PETHEMAALL93	Positive	15	0	15	0	NA	Aneuploidy
8	F	33	Pro B-ALL	NA	NA	PETHEMAALL93	Negative	59	0	54	1	MLL-AF4	del TP53

Rituximab is the first generation of anti-CD20, chimeric monoclonal antibody-based drugs. Rituximab binds to the CD20 antigen on B-cell surface, activating complement-dependent B-cell cytotoxicity, as well as to the human Fc receptors thus mediating cell killing through an antibody-dependent cellular toxicity (81–84). According to the GRAAAL 2005 trial, by adding rituximab to the standard ALL chemotherapy protocol in young adults with CD20-positive Ph-negative ALL, EFS is improved and OS after CR1is prolonged (27, 85). Other promising compounds to be used as single agent or in combination with chemotherapy are currently in different stages of clinical development. One example is ofatumumab, a second generation fully human anti-CD20 antibody (76). Initially approved for chronic lymphocytic leukemia (CLL) refractory to fludarabine (86), it targets a membrane proximal small-loop epitope on the CD20 surface marker and it was found to be more potent than rituximab in promoting complement-dependent cytotoxicity *in vitro* (87, 88). The combination of hyper-CVAD and ofatumumab is highly effective in patients with CD20-positive ALL, with 98% of patients achieving CR after the first cycle (89, 90). As follows, ofatumumab represents a potential alternative frontline therapy for CD20+ pre-B-ALL, as well as an option for patients who failed a first-line rituximab-based regimen.

Obinutuzumab is another fully humanized anti-CD20 antibody that binds to an epitope of CD20, which partially overlaps with the epitope recognized by rituximab. However, obinutuzumab is more rapid and effective than rituximab in inducing antibody-dependent cell mediated cytotoxicity, followed by direct cell death (77, 90). The drug shows promising results in trials for CD20- positive B-ALL (91–93).

## Anti-CD52 Immunotherapy

CD52 is an antigen involved in T cell activation, is expressed in 70% of T-ALL cells and pre-B ALL cells (80, 94). Alemtuzumab is a humanized monoclonal antibody against CD52. The drug was evaluated in R/R ALL, in pediatric patients and in adults (95, 96). As a single agent for pediatric ALL, alemtuzumab has limited efficacy. This anti-CD52 antibody was not eligible for further investigation due to the results obtained in a phase III trial, where in adults the combination with G-CSF exhibits clinical improvement of the disease for a shorter period of time than current treatment.

Apart from the monospecific antibodies that target one cell surface antigen or protein, modern immunology brought forward more complex designs of drugs, in which a monoclonal antibody is bound to either to a toxin or to two different cell surface proteins (97–100). Inotuzumab ozogamycin (InO) is a humanized monoclonal antibody against CD22 (inotuzumab), linked to a cytotoxic agent from the class of calicheamicin (ozogamycin) that induces double-strand DNA breaks (101). InO was studied in adults with R/R ALL and, as expected, lower response rates were observed among patients with an increased disease burden. Noteworthy were the lower response rates in patients who received InO in salvage chemotherapy after the second relapse in comparison with patients after the first relapse. Among patients with very bad prognostics, InO administration resulted in bone marrow CR rates substantially higher than in patients treated with intensive chemotherapy, although the responses were transient in the second case (102–104). Weekly *versus* single-dose clinical experience indicates that weekly InO has similar efficacy, but less systemic toxicity in comparison to single-dose administration. Despite high CR rates, the response was not durable, and the median survival was modest (5–7 months) (105). Still, the transient CR allowed 40% of patients in the InO arm to proceed to an allogeneic SCT, in comparison with the control arm, where only 17% of patients underwent through an SCT. When comparing standard therapy to InO, Kantarjian et al. showed that patients treated with InO had higher CR rates (80.7 vs. 29.4%) and MRD negativity (105). In a phase I/II clinical trial, comparing InO in combination with low-intensity chemotherapy (mini- hyper-CVAD) as frontline therapy for elderly patients with ALL, the 3-year CR and OS rates were 72 and 54% respectively. In comparison to the elderly patients treated with hyper-CVAD without rituximab, mini-hyper-CVD plus InO resulted in significantly higher 3-year OS (54 vs. 31%; *p* = 0.007). Because of the poor tolerance of these patients, anthracyclines were eliminated, whereas cyclophosphamide, prednisone, methotrexate and cytarabine were given at reduced doses. InO was administered on day 3 of every cycle (101). Thus, mini-hyper-CVD plus InO is safe and effective in elderly patients with newly diagnosed ALL, since current evidence shows that it improves outcomes when compared to hyper-CVAD.

## Anti-CD22 Immunotherapy

CD22 is a B-lineage differentiation antigen that emerged lately as a leading therapeutic target in B-ALL, due to its presence in 50–100% of adults and 90% of pediatric B lymphocytes (65, 106). Epratuzumab is an unconjugated humanized monoclonal anti-CD22 antibody introduced in phase I-III clinical trials for both adult and pediatric R/R ALL (107). For children with ALL, epratuzumab plus Hyper-CVAD chemotherapy was used for R/R ALL, with CR being obtained in 60% of cases; 46.6% of which achieved complete MRD clearance at the end of re-induction. In a phase II multicentric clinical trial for pre-B ALL in adults with R/R disease, Advani et al. evaluated the effects of adding epratuzumab to clofarabine/cytarabine therapeutic scheme and reported a superior response rate when compared to the historical data obtained in the case of single treatment with clofarabine/cytarabine (108). The internalization of epratuzumab with SN-38 is interesting as it was proven to be effective in a preclinical setting. However, clinical research is needed before we can accurately assess its efficacy (109).

Moxetumomab pasudotox is a recombinant anti-CD22 immunotoxin and a reformulation of BL22 (110, 111). Its chemical structure includes the variable region (Fv) of an anti-CD22 monoclonal antibody fused to a truncated form of *Pseudomonas aeruginosa* exotoxin A (112–115). The compound was studied in a phase I clinical trial for children with ALL, showing a manageable safety profile and evidence of activity for R/R ALL (116). For adults with adult R/R ALL, the study is still ongoing, and results have yet to be published or presented to major hematology/oncology meetings.

Combotox is a 1:1 mixture of two immunotoxins synthesized by coupling deglycosylated ricin A chain to monoclonal antibodies directed against CD22 and CD19 (117). Herrera et al. showed that the administration of Combotox in pediatric patients with R/R ALL leads to CR in 18% of cases (118–120). Patients experienced more than 95% reduction in circulating blasts in peripheral blood thus showing good promise of future clinical applications for this active compound.

## Anti-CD19 Immunotherapy

Leukemia stem cells are CD19-positive malignant lymphoblasts responsible for relapse and resistance to chemotherapy in ALL (121), this fits the principle applied to other malignancies, which states that cancer stem cells are a cellular subpopulation responsible for cancer relapse, dissemination and resistance to conventional therapy due to its high adaptability to external stressors (122–129). Topp et al. confirmed the clinical efficacy of anti-CD19 antibody in MRD-positive ALL (130). Thus, the use of combotox in this scenario might be useful in eliminating the residual non-dividing stem cells. As a single agent, combotox is useful in very heavily pretreated patients, relapsed after multiple lines of therapy (120), for which a reduction in peripheral blood count was reported in all patients. Still, in every single case the blast count rebounded after stopping the administration of combotox. Barta et al. used this molecule in combination with cytarabine in a murine preclinical model of ALL (131) and reported that the sequential administration of cytarabine and combotox was superior in comparison to therapeutic schemes. The preclinical experiments were continued in a phase I clinical trial, without any clear results in R/R ALL.

Coltuximab ravtansine (SAR3419) is an antibody-drug conjugate with humanized antibody (Coltuximab) bound to maytansin DM4, a potent cytotoxic agent (99, 132, 133). Coltuximab selectively targets CD19 antigen, present on most B cells. The binding results in the internalization of drug receptor and intracellular release of DM4 that further induces cell cycle arrest and apoptosis. SAR3419 monotherapy impairs the progression of pre-B-ALL xenografts in preclinical models. It has an objective response by delaying disease progression even in the chemotherapy-resistant xenografts models. Unfortunately, the data was not confirmed in the clinical study coordinated by Kantarjian et al. for R/R ALL (134), where out of 17 patients only 4 presented partial response (PR) with duration of only 1.9 months.

Denintuzumab mafodotin (SGN CD19A) is the second anti-CD19 conjugated monoclonal antibody, composed of a humanized anti-CD19 antibody bound to the microtubule-disrupting agent, monomethyl auristatin F. Denintuzumab is bound to monomethyl auristatin F via a maleimidocaproyl linker (117, 135–137). In a phase I study, 49 patients with R/R B-ALL or B-cell lymphoma were included and 35% achieved CR. Surprisingly, among Ph-positive B-ALL patients, the response rate was 63%, leading to increased enrolling of Ph-positive B-ALL patients for an expansion cohort. Promising results in pretreated R/R patients offer the opportunity for combination with other traditional antileukemic therapies in lymphoblastic malignancies.

Loncastuximab tesirine (ADCT-402) is the newest anti-CD19 antibody (138, 139). This humanized monoclonal antibody is conjugated, via a cleavable linker comprised of valine-alanine and maleimide, to a cytotoxic cross-linking agent (pyrrolobenzodiazepine dimer), which targets DNA minor grooves, with potential antineoplastic activity. *In vitro*, ADCT-402 showed potent cytotoxicity in a panel of human-derived cell lines of different levels of CD19, while its potency was strongly reduced in CD19-negative cell lines. *In vivo*, ADCT-402 confirmed superior anti-tumor activity when compared to both B-cell precursor ALL, under the same accelerated approval program.

## Blinatumomab for B-cell ALL

Blinatumomab is composed of two single-chain variable antibody fragments (scFv) connected by a flexible linker. One scFv binds to the CD19 antigen, which is expressed on more than 90% of B-cell cancer lineages, and the other scFv binds to the T-cell receptor/CD3 complex. Thus, blinatumomab brings B-cells and T-cells in contact, activating cytotoxic T-cells to release cytolytic proteins that induce apoptosis (140, 141). Blinatumomab only transiently engages CD3^+^ T-cells and CD19^+^ B-cells, a feature that differentiates this molecule from monoclonal antibodies. Also, preclinical experiments showed that there is no apparent target saturation and that one T-cell could engage and eliminate multiple B-cells (142).

Blinatumomab effects include CD3^+^ T-cells proliferation and activation, release of cytokines and CD19^+^ B-cells elimination. In a phase I clinical trial, after treatment initiation, redistribution of CD3^+^ T-cells produces a decline in peripheral T-cells that rapidly recover. In some patients, T-cell expansion above baseline can occur. In a dose-dependent relationship, a rapid decline in B-cells was observed after treatment initiation. Some patients with R/R ALL may not respond to treatment and present with unchanged or marginally decreased B-cell levels. Cytokines such as IL-10, IL-6 and IFN-γ may increase rapidly after treatment initiation. Cytokine release may be dose-dependent, with higher levels at higher doses and may also be influenced by tumor load (CD19^+^ B-cell count) (141–143). The pharmacokinetics (PK) of blinatumomab is similar to that of other small proteins, considering that it undergoes rapid clearance from the systemic circulation via catabolism. Blinatumomab exhibits first-order elimination kinetics, meaning that after the infusion is stopped, plasma concentrations decrease rapidly. Drug clearance was found to be fast and elimination half-life short.

Because blinatumomab is not glycosylated, it has a short half-life. The introduction of glycans reduces clearance due to their negative charged sialic acids and their size. These characteristics impair cell membrane transfer for catabolism and renal filtration. Blinatumomab is not subjected to hepatic metabolism and does not influence CYP enzymes activity. However, blinatumomab can increase IL-6 levels, a cytokine with inhibitory activity against CYP enzymes (143)_._

Multiple clinical trials have been conducted to test the efficacy and safety of blinatumomab for R/R ALL. The first two clinical trials on blinatumomab, MT103-206 and MT103-211 evaluated blinatumomab pharmacokinetics, efficacy, safety, and tolerability in adults with Ph-negative ALL and were at the basis of the marketing authorization. The regular FDA approval in 2014 was based on the results of a phase III trial (TOWER), which demonstrated a superior OS for blinatumomab in comparison to standard-of-care chemotherapy in patients with R/R Ph-negative ALL. Blinatumomab- treated patients had a median OS of 7.7 vs. 4.0 months in the case of R/R Ph- ALL patients treated with standard chemotherapy. Additionally, the efficacy of this drug for the treatment of R/ R Ph-positive ALL was evaluated in the single-arm trial, ALCANTARA, which showed CR in 31% patients, with a median CR duration of 6.7 months. The results of the ALCANTARA trial laid the basis for the expansion of blinatumomab indications, which now include both Ph-negative and Ph-positive R/R ALL (144). MT103-205 was a clinical trial with blinatumomab in pediatric and adolescent patients with R/R ALL, which showed a CR rate of 17% and a median OS of 8 months, hence contributing to the decision to grant full approval of blinatumomab for the treatment of children over 1 year of age and adolescents (145–147). Recently, based on the results of BLAST, a phase II study evaluating blinatumomab efficacy and safety for the treatment of patients in remission with minimal residual disease (MRD), blinatumomab indications were extended to include this patient population. BLAST identified a 78% complete MRD response after 1 treatment cycle. Across all treatment cycles complete MRD was achieved by 80% of patients (148). A recent meta-analysis that included 7 studies, with a total of 708 R/R ALL patients identified a CR rate between 0.36 and 0.69, with a pooled CR rate of 0.45, reassuring blinatumomab effectiveness in R/R ALL. An important influence over CR rate had the tumor load before blinatumomab treatment, ranging from 0.75 CR for <50% bone marrow blast percentage, to 0.33 CR for patients over 50% tumor load, underlining the usefulness of chemotherapy before blinatumomab for reducing tumor load. Also, blinatumomab presented a pooled MRD response rate of 0.42, suggesting efficiency in eliminating MRD. After the blinatumomab treatment, a total of 148 (23.6%) patients were subjected to allogeneic HSCT, indicating blinatumomab treatment is an important step for successful transplantation. Preliminary data suggests blinatumomab is effective in R/R Ph+ ALL, with a 35.6% CR rate. Moreover, as single agent, blinatumomab obtained a 6–10-month OS and a 5–8-month RFS, important contributors to MRD (149).

In clinical trials, blinatumomab presented mostly adverse effects (AEs) that correlated with its mechanism of action. CRS, neurotoxicity, and hypogammaglobulinemia were among the most frequent AEs (145–147, 149, 150). CRS usually develops after IV administration of immunotherapy, upon release of inflammatory mediators and cellular cytokines into the systemic circulation. This systemic inflammatory response may affect cardiovascular, renal, respiratory, and neurologic function. CRS can be life-threatening but, in most cases, symptoms are mild. It occurs mostly in the first days of the first and second cycle of treatment, most often in patients with a high tumor burden (8, 9). Treatment of CRS consists of blinatumomab temporary withdrawal and high dose steroids (dexamethasone). Prevention consists of dose up-titration (9 μg/day for the first week of the first cycle, followed by 28 μg/day, for the rest of the cycle) and dexamethasone pre-medication before treatment initiation and dose increase (17, 151, 152). Neurological toxicity is a relatively frequent AE, with an increased incidence in older patients (more than 65 years, 72%). It is believed to be caused by T-cells binding CD19-positive B-cells in the CNS and subsequent cytokine release, leading to inflammation and an increase in blood-brain-barrier permeability. Neurotoxicity includes events such as: seizures, irritability, disorientation, tremor, and encephalopathy. Treatment consists of blinatumomab withdrawal sometimes coupled with administration of steroids and anticonvulsants (130, 153, 154). Another important AE, infections (sepsis and pneumonia), was observed in 25% of patients in clinical studies. The most important cause of infection is B-cell depletion and decreased concentrations of immunoglobulins. Prevention of infections may be performed with prophylactic anti-infective therapies during blinatumomab treatment (144).

The TOWER and ALCANTARA trials revealed several advantages of blinatumomab over SOC chemotherapy. Cytopenias, such as neutropenia and related infections occurred less frequently in patients receiving blinatumomab. However, CRS, pyrexia, tremor and encephalopathy were more common among patients receiving blinatumomab. Also, depression was identified as a new adverse reaction, with potentially severe consequences and presumed to be caused by neurological toxicity (144). Medication errors have been reported with the use of blinatumomab due to the complex method of preparation and administration. Overdoses or underdoses resulted from preparation errors, from miscalculations or by malfunctions of the infusion pump. In the phase II study, MT103-211, overdoses were reported for 3% of patients, with symptoms including fever, tremors and headache. Overdose treatment includes blinatumomab withdrawal, patient monitoring and supportive care. To avoid medication errors, product label includes comprehensive instructions for preparation, administration and a warning underlying the importance of strictly following the label instructions in order to avoid medication errors (145).

In patients transplanted after blinatumomab therapy, the team of Handgretinger et al. report that the major toxicities include seizures and cytokine release syndrome (155). Treatment based on blinatumomab has shown impressive efficacy, but it associated with important yet manageable toxicity (130, 154, 156). Patients sometimes have a transient cytokine release immediate after initiation of therapy and develop flu-like symptoms, fever or headaches (8, 9). The main cytokines involved are interleukin (IL)-10, IL-6 and interferon gamma, also described to be linked to hemophagocytic lymphohistiocytosis (HLH). HLH may be primary and is determined by various germline mutations involved in cytolytic granule exocytosis and allows spontaneous macrophage activation after a minimal trigger, or it can be secondary, known as macrophage activation syndrome (157). Macrophage activation syndrome is triggered by infections, autoimmune disorders or by an underlying malignancy (158, 159). Neurotoxicities include even life-threatening generalized cerebral seizures with apnea, treated immediately with intravenous lorazepam and followed by antiepileptic prophylaxis with leviracetam and dexamethasone (83, 160). A brain CT scan is indicated after the acute seizure (161, 162). Deaths related to graft-vs.-host disease were not reported (163, 164), but infections may occur and should be managed according to the European Conference on Infections in Leukemia (ECIL) guidelines (165).

Considering the risks associated with blinatumomab administration, both FDA and EMA have issued risk management plans that include pharmacovigilance activities and a comprehensive product label informing about possible risks, specific monitoring, prevention, and management. Blinatumomab is currently under continuous post-marketing monitoring in order to identify possible additional risks associated with its use (166–168).

## How to Treat MRD-positive R/R B-cell ALL with Monoclonal Antibodies in Developing Countries: The Real-life Setting in Romania

Clinical experience in the real-life setting is often different from the phase III clinical trial that preceded the approval of a drug by the FDA or other regulatory agencies (162, 169). Modern medicine addresses the real-life experience as it influences both the economics of a healthcare system, as well as the outcome of therapy. We chose to present the situation in Romania, as this country is meaningful for the standard scenario of a developed country, part of the Western world, but often not directly involved in the design and coordination of state-of-the-art clinical trials in cancer immunotherapy. A Romanian patient is often the “target” patient, that is treated in a healthcare system that can afford to be reimburse the treatment, but is still not in an ideal setting, as it's similar cases from Western Europe or the US. Romania's experience may be extrapolated for other healthcare systems, as is the case for Eastern Europe, South American or developed Asian economies. Thus, one of the aims of the current manuscript is to present the real-life experience of blinatumomab in Romania, 2 years after its approval by the FDA. Thus, we present in a clinical scenario a standard relapsed B-cell patient, as well as the cohort of Romanian patients treated with this novel therapy.

**Figure 2:**
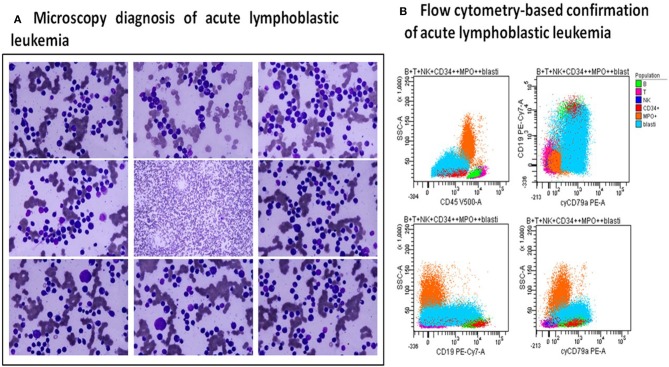
Diagnosis of B-cell ALL.

A 24-year old male presented to consultation with the complaints of bilateral laterocervical lymph nodes, dysphagia, cough, mild fever, and weight-loss for the past weeks, but no remarkable medical history. Physical examination revealed laterocervical, submandibular, axillary and inguinal lymph nodes (of about 2 cm) and splenomegaly (2 cm below the costal margin). The blood examination noted leucopenia (3.14 × 10^3^/μl) with 22% blasts in the peripheral blood, and the bone marrow aspirate found 60% blastic infiltrate. The immunophenotyping analysis revealed a common ALL (CD45+, CD19+, CD22+, CD10+, HLA-DR+, CD34+, CD33+) (Figure 2).

Standard chemotherapy using the Hyper-CVAD protocol was initiated, obtaining complete hematological response after the first cycle (A+B). In December 2013 the patient was MRD positive, thusly treatment with blinatumomab was initiated in January 2014. In July, after four cycles of blinatumomab, complete molecular response was obtained, and the POMP therapy was administered as maintenance until December 2016. Unfortunately, in July 2017, the patient relapsed: the hematologic exam revealed anemia (hemoglobin = 9.6 g/dl), mild thrombocytopenia (103 × 10^3^/μl) and 22% blasts. Under these circumstances, chemotherapy was reinitiated, following the HyperCVAD protocol, and a second complete hematologic remission was obtained. At this time, family was tested for HLA compatibility, without positive result: patient was put on the waiting-list for matched unrelated allogeneic SCT. With the approval of the Romanian National Drug Agency, the therapy with blinatumomab was reinitiated in December 2017. At the moment, after administration of 2 cycles of blinatumomab, patient is in remission, waiting for a compatible donor for an allogeneic SCT. The treatment with blinatumomab was well-tolerated, patient presented some fever spikes and grade 1 neurological adverse events, that could be controlled with the administration of corticosteroids. It is worth mentioning that severe polyclonal hyperglobulinemia was present even at 4 years after the first blinatumomab treatment (IgG=123 mg/dl, normal value 700–1,600 mg/dl), but with a low rate of infectious complications. Afterwards, the patient proceeded to an allogeneic SCT and is now in CR.

**Figure 3:**
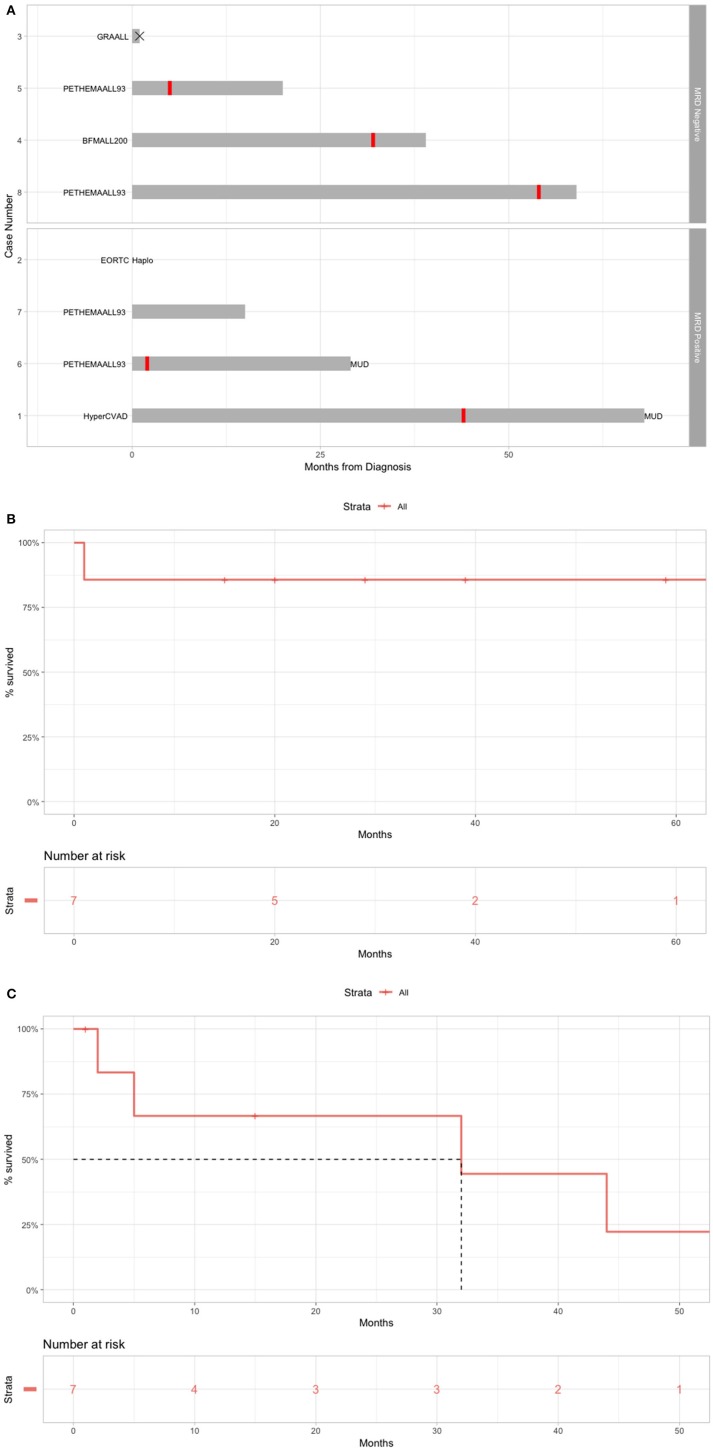
Real-life setting data on the use of blinatumomab for B-cell ALL. **(A)** Overall treatment of the patients before blinatumomab. **(B)** OS for patients treated with blinatumomab in Romania. **(C)** RFS for patients treated with blinatumomab in Romania.

The Romanian experience with R/R B-cell ALL patients treated with blinatumomab is presented in Table 1. The B-cell ALL patient evolution is presented in Figure 3A, the OS is presented in Figure 3B and the RFS is presented in Figure 3C.

## Conclusion

From the development of rituximab, several antibody-based therapeutic options for B-ALL were developed. These are targeted against B-cell antigens, such as CD22, CD20, CD19. The anti-B cell antibodies can be conjugated with a cytotoxic agent, and lead to direct cell death or another antibody against T-cell antigens: CD52 or CD3, followed by indirect cytotoxic T cell death. Blinatumomab is a double antibody against CD19 and CD3 containing only the Fc region of antibody, thus establishing only a temporary link between normal cytotoxic T cells and malignant B cells. This offers higher therapeutic efficiency to the drug and lower the side effects. The most severe side effect being the cytokine re Despite development in ALL treatment, most of patents relapse after CR. We presented a sequence of eight B-ALL clinical cases treated with blinatumomab. Our experience showed that blinatumomab has a good tolerability and a great efficacy even in the case of R/R ALL. With the proof-of-concept sequence of case reports show that blinatumomab is efficient and with good outcome, that may provide a useful insight into improving the development of R/R B-ALL immunotherapy.

The major issue of this manuscript is not to present modern therapeutics for B-cell ALL, but to present the real-life situation of monoclonal antibody-based drugs in Eastern European Union. We present Romania's situation as a proof-of-concept in the last chapter and present the classic “How I treat” scenario to the lea-life setting of 2019–2020. Monoclonal antibodies have changed the face of cancer immunotherapy and will continue to do so. Thus, we stress out the importance of access to such drugs, may it be in the clinical trial setting, or following approval and reimbursement by the national drug agencies. Much progress was made in the last 10–15 years, but much more is needed, with the major goal of providing the leukemia patient with state-of-the-art treatment protocols and achieve disease long-term remission.

Thus, the present manuscript is not a systematic review and should be regarded as a classic review, with all its limitations, in which we present the novel insights of bispecific antibodies in R/R B-cell ALL, as well as in the last chapter the real-life experience with blinatumomab for these patients.

## Data Availability Statement

All datasets generated for this study are included in the article/supplementary material.

## Author Contributions

All authors contributed in the clinical management of the patients. DD wrote the manuscript. CT and HE supervised the manuscript.

### Conflict of Interest

The authors declare that the research was conducted in the absence of any commercial or financial relationships that could be construed as a potential conflict of interest.
